# Long-term effects on the progress of neuropathy after diabetic Charcot foot: an 8.5-year prospective case–control study

**DOI:** 10.1186/s13104-018-3253-5

**Published:** 2018-02-20

**Authors:** Rasmus Bo Jansen, Tomas Møller Christensen, Jens Bülow, Lene Rørdam, Per E. Holstein, Ole Lander Svendsen

**Affiliations:** 1 Copenhagen Diabetes Foot Center (CODIF), Bispebjerg Hospital, University of Copenhagen, 2400 Copenhagen NV, Denmark; 2 Department of Endocrinology, Bispebjerg Hospital, University of Copenhagen, 2400 Copenhagen NV, Denmark; 3 Department of Clinical Physiology and Nuclear Medicine, Bispebjerg Hospital, University of Copenhagen, 2400 Copenhagen NV, Denmark; 4 Copenhagen Wound Healing Center, Bispebjerg Hospital, University of Copenhagen, 2400 Copenhagen NV, Denmark

**Keywords:** Diabetes, Neuropathy, Charcot foot, Follow-up

## Abstract

**Objective:**

Charcot foot is a severe complication to diabetes mellitus, associated with diabetic neuropathy. Any long-term effects of a Charcot foot on the progress of neuropathy are still largely unexplored. The objective was to investigate whether a previous Charcot foot had any long-term effects on the progress of neuropathy.

**Results:**

An 8.5-year follow-up case–control study of 49 individuals with diabetes mellitus, 24 of whom also had Charcot foot at baseline visit in 2005–2007. Neuropathy was assessed with a questionnaire, biothesiometry, heart rate variability and venous occlusion plethysmography. Of the 49 baseline participants, 22 were able to participate in the follow-up. Twelve had passed away in the meantime. Heart rate variability was unchanged in both groups; from 9.7 to 7.2 beats/min (p = 0.053) in the Charcot group, and 14.3 to 12.6 beats/min (p = 0.762) in the control group. Somato-sensoric neuropathy showed no difference between baseline and follow-up in the Charcot group (from 39.1 to 38.5 V) (p = 0.946), but a significantly worsened sensitivity in the control group (from 25.1 to 38.9 V) (p = 0.002). In conclusion, we found that any differences in somatic or cardial autonomic neuropathy present at baseline had disappeared at follow-up after 8.5 years.

**Electronic supplementary material:**

The online version of this article (10.1186/s13104-018-3253-5) contains supplementary material, which is available to authorized users.

## Introduction

Charcot foot (CF) is an unusual bone and joint disorder which causes hyperemia, inflammation and degeneration of the weight-bearing structures in the foot. If left untreated it can result in spontaneous fatigue bone fractures, leading to deformity and ulcerations [1, 2]. Although the precise pathological mechanism underlying the Charcot foot is unknown, it is exclusively seen in individuals with established peripheral neuropathy [3–5]. Most cases in the Western world occur in patients with diabetes mellitus, with an incidence rate of about 0.3% per year [6, 7].

We have previously shown that local autonomic neuropathy in the feet, assessed through a weakened venoarterial sympathetic axon reflex, is not directly related to the development of an acute Charcot foot, despite the increased blood flow [8] (compared to diabetic controls with and without somato-sensoric neuropathy). Furthermore, there seemed to be a dissociation between local and central autonomic neuropathy (as measured by heart rate variability through beat-to-beat analysis). This supports the theory that the inflammation is provoked by repeated local microtrauma and bone remodelling, as suggested by others [9–12].

It is still unknown whether the chronic deformities from a Charcot foot are related to lasting alterations in systemic or local neuropathy. If so, that could account for some of the recurrences of Charcot foot which are so far somewhat unexplained [13, 14].

To investigate this we conducted a follow-up study of a previously well-described group of diabetes patients with or without a Charcot foot [8, 15, 16]. The main goal was to investigate if a former Charcot foot had any long-term effects on somato-sensoric or autonomic neuropathy, including local blood flow.

## Main text

### Methods

A total of 49 patients were included at the baseline visit in 2005–2007 [8, 15]. All 49 patients were followed up using medical records, the “Danish Register of Cause of Death” and the “Danish Civil Registration System”. The included patients were 61.7 ± 7.2 years old and were distributed as 40 males and 9 females. Ten patients had type 1 diabetes, and 39 had type 2 diabetes.

At baseline in 2005–2007 all examination were performed by author TMC, and at follow-up by author RBJ. All examinations were performed using the same equipment and the same procedures. Patients were examined in order of their availability over an 11 months period at the Endocrine Research Unit at Bispebjerg Hospital, Denmark.

Physical examinations focused on the lower extremities including deformities, wounds and loss-of-function. The degree of neuropathy was assessed by the modified questionnaire Neuropathy Symptom Score (NSS) (Additional file 1: Appendix S1) [17, 18]. In addition, vibration perception thresholds was measured by biothesiometry (Biothesiometer, Bio-Medical Instruments Co, Newbury, Ohio 44065, USA), with the probe placed on the hallux pulpa [19–21].

Cardial Autonomic Neuropathy (CAN) was assessed using the heart rate variability method by utilizing the autonomic cardiac regulation. Measurements were done using PowerLab, Chart 5 v5.5 (ADInstruments Pty Ltd, Castle Hill, NSW, Australia). For electrocardiography measurement three electrodes were placed on the patient’s chest. The patient was instructed to rest for 10 min, after which baseline readings were taken. The patient was then instructed to adjust their respiration rate to 6 steady breaths per minute for 1 min, each inspiration and expiration phase taking 5 s. This was controlled with a stopwatch and under constant instruction and monitoring. The procedure was repeated five times with 5 min of rest between measurements. The measurements selected as results were the ones which were evaluated to display the best compliance to the instructions (based on respiration and heart-rate pattern) [22].

Blood flow in the feet was measured using venous occlusion plethysmography [23, 24] with a Hokanson EC6 Plethysmograph with a Hokanson E20 Rapid Cuff Inflator (Bellevue, WA, USA). The measurements were done with the patient lying on his/her back after a rest period of 30 min. The cuff was placed around the calf, and a fitting strain gauge was placed around the forefoot. Cuff pressure was 40 mmHg for 7 s, and the inflation was repeated 5 times on each foot.

Data are expressed as means ± 1 Standard Deviation (SD) unless otherwise noted. Normal distribution was controlled with the Shapiro-Wilks tests. T-tests and Mann–Whitney rank sum tests were used for comparisons between two data sets (parametric and non-parametric respectively).

For variance analysis between multiple ranked groups the non-parametric Kruskal–Wallis one way analysis of variance was used. For matched groups (e.g. baseline versus follow-up) paired t-tests and Wilcoxon signed rank tests were used. For compairing categorical data (2 × 2 tables), Chi square test was used. Results were adjusted using Bonferroni correction when applicable (multiple comparisons).

Statistics and generel data handling was done using IBM SPSS Statistics v. 23 by IBM Corporation, SIGMAPLOT v. 11.0.0.77 by Systat Software Inc., Microsoft Excel 2000 v. 9.0.2812 by Microsoft Corporation and Apache OpenOffice 4.0.1 by The Apache Software Foundation.

### Results

Of the 49 patients at the baseline visit, 12 had passed away (Table 1 and Additional file 2: Figure S1). Thus, the mortality rate over 8.5 years was about 25%. The causes of death were mainly related to late stage diabetic complications, and were: nephropathy (n = 3), cancer (n = 3), coronary artery disease (n = 2), complicated infections/septicaemia (n = 2), postoperative pulmonary embolism (n = 1) and unknown (n = 1).

**Table 1:** Distribution of participants (all with diabetes) at baseline visit in 2005–2007, and distribution and cause of not participating at follow-up visit in 2015

	Groups with diabetes and Charcot foot		Groups with diabetes but without Charcot foot		
Distribution of participants at baseline visit 2005–2007^a^	Acute CF (n = 17)	Chronic CF (n = 7)	1. toe amputee (n = 5)	Neuropathy (n = 9)	Without neuropathy (n = 11)
Passed away before follow-up	3 (17%)	3 (43%)	3 (60%)	3 (33%)	0 (0%)
How many were amputated before death	2	2	1	0	N/A
Unable to reach/moved abroad	1	1	1	0	1
Declined participation	2	0	1	2	2
Excluded due to amputation	1	2	0	1	0
Participants available for follow-up measurements	10	1	0	3	8
Distribution of participants at follow-up visit 2015	Previous Charcot foot (DM + CF) n = 11		No previous Charcot foot (DM − CF) n = 11		

^a^Please see [11, 18] for details

We were unable to reach 4 former participants, 3 of whom had moved abroad. Of the remaining 33, 7 didn’t want to participate while 4 had had some form of lower extremity amputation due to diabetic foot ulcers. In total, 22 of the original 49 patients were able to participate in some or all of the follow-up examinations, giving the follow-up visit a participation rate of 45%.

Due to the high loss-to-follow up the participants were pooled into two groups: Diabetes mellitus with or without previous Charcot foot. This distributed the participants in two even groups of 11 each. The group with diabetes and previous Charcot foot is labeled as “DM + CF”, and the group with diabetes but without previous Charcot foot is labeled as “DM–CF”. Antropomorphic data on the participants at the follow-up visit are listed in Table 2.

**Table 2:** Antropomorphic data of the participants at follow-up for both groups of diabetics with (DM + CF) or without (DM − CF) previous Charcot foot

Group	DM + CF (n = 11)^a^	DM − CF (n = 11)^b^
Age (years)	67.2 ± 8.3	70.4 ± 3.8
Sex (m/f)	8/3	10/1
Diabetes type (I/II)	5/6	2/9
Diabetes duration (years)	28.0 ± 15.4	26.7 ± 12.8
Affected foot (right/left)	6/5	N/A
BMI	27.2 ± 3.2	30.0 ± 4.8
HbA1c (mmol/mol)	59.9 ± 10.2	58.3 ± 12.8

Data listed as #n or mean ± 1SD. None of the parameters are significantly different between the groups

^a^Group represents pooled data from acute and chronic Charcot foot groups from the first study [11, 18]

^b^Group represents pooled data from 1.toe amputees, neuropathy and without neuropathy groups from the first study [11, 18]

Data was tested for any possible skewering from a survivor bias due to the high mortality. We pooled the participants with former CF (acute and chronic), and those without to test them in a 2 × 2 table. There was no significant difference in mortality between the two groups (p = 0.863). We divided the baseline population into follow-up (n = 22) and non follow-up participants (n = 27) and tested the two groups against each other for variations regarding age, diabetes age, BMI, HbA1c or biothesiometry without any significant finds (Additional file 3: Table S1).

None of the patients had had a subsequent reactivation of their Charcot foot since the baseline visit. None had active foot ulcers at follow-up either, though some patients in both groups reported having one or more foot ulcers in the follow-up period (n = 6 in the DM + CF group and n = 3 in the DM − CF group).

In the Charcot group, 4 patients had visual foot deformities (assessed without x-ray) after their Charcot foot. All patients with CF were originally treated with off-loading in a removable off-loading cast, worn for an average of 7.7 months (range 16.0 and median 6.0), and all were subsequently provided with some form of special off-loading shoe wear.

The modified NSS at follow-up showed a mean of 3.1 points for all 22 participants, which was not significantly different from the 2.4 points at baseline. Neither the DM + CF nor the DM – CF groups showed a significant difference from baseline to follow-up either (Table 3).

**Table 3:** Somato-sensoric and autonomic neuropathy in the groups from baseline and follow-up

	DM + CF (n = 11)	DM − CF (n = 11)	*p* value
Mod.NSS, baseline	2; 8	1; 6	0.946
Mod.NSS, follow-up	4; 7	3; 9	–
p value	0.250	0.557	–

	DM + CF (n = 11)		DM − CF (n = 11)		p value^+^
	Healthy foot	Charcot foot	Right foot	Left foot	
Biothesiometry, baseline (V)	38 ± 15	40 ± 15	26 ± 11	24 ± 13	0.014*
Biothesiometry, follow-up (V)	38 ± 15	39 ± 15	40 ± 10	38 ± 10	–
Δ_follow-up_	− 1 ± 11	− 1 ± 5	14 ± 13	14 ± 16	0.003*

	DM + CF (n = 11)	DM − CF (n = 6)	p value
Heart-rate variability, baseline (beats/min)	9.7 ± 5.9	14.3 ± 7.5	0.127
Heart-rate variability, follow-up (beats/min)	7.2 ± 4.0	12.6 ± 7.7	–
p value	0.053	0.762	–
Δ_follow-up_	− 2.5 ± 3.7	− 0.4 ± 3.2	0.274

Groups consists of diabetics with (DM + CF) or without (DM − CF) acute or chronic Charcot foot at baseline

Data listed as (median; range) or (mean ± 1SD)

* Significant at set level (α-level) of 0.05

^+^Average of both feet in each group (DM + CF and DM − CF) compared

The biothesiometry measurements at baseline showed a significant difference between the average sensitivity in the DM + CF and the DM − CFgroups, with the DM + CF group’s feet having a higher vibration threshold than the DM − CF group’s feet (p = 0.014). The change in sensitivity from baseline to follow-up was significantly increased for the DM − CFgroup compared to the DM + CF group (p = 0.003), to a degree that the two groups had comparable vibration thresholds at follow-up.

CAN was measured in 17 of the 22 participants; 2 wished not to participate while 3 other procedures were unfit for analysis due to a pacemaker (n = 1) or the participants’ inability to cooperate to the measurements (n = 2).

There was no difference in the heart rate variability at baseline between the DM + CF and the DM − CF groups (p = 0.127). The change from baseline to follow-up was unchanged both within the groups and between them (Table 3). In the DM + CF group (n = 11) it changed from 9.7 ± 5.9 to 7.2 ± 4.0 beats/min (p = 0.053), and in the DM − CF group (n = 6) it changed from 14.3 ± 7.5 to 12.6 ± 7.7 beats/min (p = 0.762).

Of the 22 participants, 19 were able to participate to the plethysmography; 1 did not wish to participate while 2 were unable to complete the procedure due to discomfort from the cuff (n = 1), or involuntary muscle cramps which invalidated the measurements (n = 1).

In the DM + CF group (n = 10) there was a significant decrease in blood flow in the previous Charcot foot between baseline and follow-up (p = 0.001), as well as a significant difference in the change from baseline to follow-up between the healthy and affected foot (p = 0.002) (Additional file 4: Figure S2).

There was no difference at follow-up between the blood flow in the DM + CF and DM − CF groups (p = 0.280). However, there was a significant change in the average blood flow in the DM + CF feet compared to the DM − CF from baseline to follow-up (p = 0.009), due to a decrease in blood flow in the DM + CF feet.

### Discussion

In this study we have described a population with diabetes mellitus, half of whom had a Charcot foot 8.5 years ago. We have focused on the possible consequences of this on peripheral sensoric neuropathy, CAN and blood flow in the foot. To our knowledge, this is the first study investigating a population with Charcot foot across those parameters long-term.

We found no differences in somato-sensoric or central autonomic neuropathy had developed during the follow-up period, and that the difference in somatic neuropathy present at baseline had disappeared at follow-up. Baseline data showed a significant difference in both sensitivity and blood flow between the groups, but this had also disappeared at follow-up.

The worsening in sensitivity was only seen in the DM − CF group. The sensitivity in the DM + CF group did not change during the follow-up period, and neither did it differ between the healthy and the affected foot. This suggests that a foot developing an acute Charcot condition is affected equally by neuropathy as the contralateral foot. Overall, there seems to be no lasting effects of an acute Charcot foot on the progress of peripheral somato-sensoric neuropathy or CAN in our population after 8.5 years.

In our population, we see a high mortality in several of the baseline groups (Table 1) which is in line with previously reported mortality rates. As shown by Sohn et al. [25] people with diabetes have an increased mortality when affected by Charcot foot and/or foot ulcers; both of which are commonly related to neuropathy. Especially diabetic neuropathic foot ulcers are known to be a major cause of mortality. Moulik et al. [26] report a 5-year mortality of 45%, while Morbach et al. report a cumulative 10-year mortality for people with diabetic foot ulcers of 70% [27].

Our main findings are that there were no differences between the previous Charcot foot group and control group with regards to somato-sensoric or autonomic neuropathy at follow-up. In fact neuropathy in participants with diabetes but without Charcot foot had significantly worsened from baseline to follow-up, to be comparable to that of those participants with previous acute Charcot foot. In addition, the increased blood flow seen in the acute Charcot foot had, as expected, completely disappeared at follow-up 8.5 years later. Finally, our study confirms the high mortality for individuals with diabetic neuropathy.

## Limitations

The main weakness of the study was the low participation rate in a small population. However, significant dropout and loss-to-follow up is often the case when dealing with patients with a serious disease and its complications. Due to the considerable loss-to-follow up, we chose to include all participants in as many tests as possible, even those who could not participate in all the tests.

## Additional files

**Additional file 1: Appendix S1.** The modified Neuropathy Symptom Score. The questionnaire used to assess patients’ symptoms of neuropathy.

**Additional file 2: Figure S1.** Flowchart of Participants. The flow of participants into the study from baseline to follow-up.

**Additional file 3: Table S1.** Data comparison between follow-up and lost-to-follow-up. Comparisons between baseline values in the population of former participants lost to follow-up, and participants who joined both baseline and follow-up measurements.

**Additional file 4: Figure S2.** Foot blood-flow. Blood-flow as measured by venous occlusion plethysmography at baseline and at follow-up 8.5 years later for participants with diabetes and an acute or chronic Charcot foot at baseline.

## Abbreviations

CAN: cardial autonomic neuropathy
CF: Charcot foot
DM + CF: diabetes mellitus and Charcot foot
DM − CF: diabetes mellitus without Charcot foot
NSS: Neuropathy Symptom Score
SD: standard deviation
