# Supplementary material for: Long-term effects on the progress of neuropathy after diabetic Charcot foot: an 8.5-year prospective case–control study
Source: BMC Res Notes. 2018 Feb 20;11:140. doi: 10.1186/s13104-018-3253-5 (PMC5819300; doi:10.1186/s13104-018-3253-5)
Supplement: Supplementary file 1 — Additional file 1: Appendix S1. The modified Neuropathy Symptom Score. The questionnaire used to assess patients’ symptoms of neuropathy. [file 13104_2018_3253_MOESM1_ESM.doc]

**Appendix S1**

**The modified Neuropathy Symptom Score**

Each item grants 1 point if present during the day, 2 points if it's exacerbated during the night, and 0 points if not present at all. Maximum score is 14. All questions are targetted at the lower extremities.

Do you experience any regular:

1. Muscle cramps?
2. Numbness?
3. Abnormal hot or cold sensation?
4. Tingling sensation?
5. Burning pain?
6. Aching pain?
7. Irritation by clothes or bed cloth?
