# Supplementary material for: Long-term effects on the progress of neuropathy after diabetic Charcot foot: an 8.5-year prospective case–control study
Source: BMC Res Notes. 2018 Feb 20;11:140. doi: 10.1186/s13104-018-3253-5 (PMC5819300; doi:10.1186/s13104-018-3253-5)
Supplement: Supplementary file 2 — Additional file 2: Figure S1. Flowchart of Participants. The flow of participants into the study from baseline to follow-up. [file 13104_2018_3253_MOESM2_ESM.doc]

**Figure S1:**

**Figure S1:** Flowchart of the participants' inclusion from the baseline to the follow-up study.
