# Supplementary material for: Long-term effects on the progress of neuropathy after diabetic Charcot foot: an 8.5-year prospective case–control study
Source: BMC Res Notes. 2018 Feb 20;11:140. doi: 10.1186/s13104-018-3253-5 (PMC5819300; doi:10.1186/s13104-018-3253-5)
Supplement: Supplementary file 3 — Additional file 3: Table S1. Data comparison between follow-up and lost-to-follow-up. Comparisons between baseline values in the population of former participants lost to follow-up, and participants who joined both baseline and follow-up measurements. [file 13104_2018_3253_MOESM3_ESM.doc]

**Table S1: Comparisons between baseline values in the population of former participants lost to follow-up,**

**and participants who joined both baseline and follow-up measurements.**

|  | **Baseline Charcot foot group (n=24)** | | | **Baseline group without Charcot foot (n=25)** | | |
| --- | --- | --- | --- | --- | --- | --- |
|  | **Follow-up participants**  **(n=11)** | **Non-participants**  **(n=13)** | **P-values** | **Follow-up participants**  **(n=11)** | **Non-participants**  **(n=14)** | **P-values** |
| **Age (year)** | 59 ±8 | 61 ±8 | 0.496 | 62 ±4 | 64 ±7 | 0.566 |
| **Diabetes age (year)** | 19 ±15 | 17 ±10 | 0.771 | 18 ±13 | 17 ±15 | 0.784 |
| **BMI (kg/m^2^)** | 28 ±2 | 32 ±6 | 0.082 | 30 ±6 | 28 ±4 | 0.209 |
| **HbA1c (mmol/mol)** | 63 ±17 | 65 ±20 | 0.805 | 63 ±14 | 62 ±17 | 0.879 |
| **Biothesiometry (V)** | 39 ±15 | 48 ±4 | 0.146 | 25 ±11 | 36 ±15 | 0.092 |
| **HRV (beats/min)** | 9.7 ±5.9 | 10.9 ±7.1 | 0.650 | 14.2 ±7.5 | 11.5 ±6.6 | 0.311 |
| **HRV^+^ for participants who were deceased at follow-up** | N/A | 8.8 ±4.5 | N/A | N/A | 13.1 ±9.3 | N/A |
| **HRV^+^ for participants lost to follow-up for other reasons** | N/A | 11.3 ±9.1 | N/A | N/A | 8.8 ±4.8 | N/A |
| **Blood-flow^#^ (mL/(100g*min))** | 4.7 ±2.4 | 4.6 ±2.2 | 0.872 | 3.1 ±1.8 | 3.7 ±2.4 | 0.526 |
| **Blood-flow^#^ for participants who were deceased at follow-up** | N/A | 6.1 ±2.0 | N/A | N/A | 4.9 ±3.1 | N/A |
| **Blood-flow^#^ for participants lost to follow-up for other reasons** | N/A | 3.2 ±1.3 | N/A | N/A | 2.7 ±1.3 | N/A |

Data listed as mean ±1SD.

+ = Heart-Rate Variability as measure for CAN.

# = Distal blood-flow as measured by venous occlusion plethysmography, both feet averaged.
