# Supplementary material for: Long-term effects on the progress of neuropathy after diabetic Charcot foot: an 8.5-year prospective case–control study
Source: BMC Res Notes. 2018 Feb 20;11:140. doi: 10.1186/s13104-018-3253-5 (PMC5819300; doi:10.1186/s13104-018-3253-5)
Supplement: Supplementary file 4 — Additional file 4: Figure S2. Foot blood-flow. Blood-flow as measured by venous occlusion plethysmography at baseline and at follow-up 8.5 years later for participants with diabetes and an acute or chronic Charcot foot at baseline. [file 13104_2018_3253_MOESM4_ESM.doc]

**Figure S2:**

**Figure S2:** Blood-flow as measured by venous occlusion plethysmography at baseline and at follow-up 8.5 years later for participants with diabetes and an acute or chronic Charcot foot at baseline.

Boxes represent 25^th^ and 75^th^ quartile, whiskers 10^th^ and 90^th^ percentile, dots are outliers.

There was a significant difference in blood flow in the Charcot foot between baseline and follow-up (6.7 versus 1.4)(p=0.001).

There was no significant difference in blood flow in the healthy foot between baseline and follow-up (2.7 versus 1.5)(p=0.148).
